# Pain Perception and Migraine

**DOI:** 10.3389/fneur.2018.00576

**Published:** 2018-08-02

**Authors:** Antonio Russo, Gianluca Coppola, Francesco Pierelli, Vincenzo Parisi, Marcello Silvestro, Alessandro Tessitore, Gioacchino Tedeschi

**Affiliations:** ^1^Department of Medical, Surgical, Neurological, Metabolic, and Aging Sciences, Headache Center, University of Campania “Luigi Vanvitelli, ”, Naples, Italy; ^2^MRI Research Center SUN-FISM, University of Campania “Luigi Vanvitelli,”, Naples, Italy; ^3^Research Unit of Neurophysiology of Vision and Neuro-Ophthalmology, G. B. Bietti Foundation-IRCCS, Rome, Italy; ^4^Department of Medico-Surgical Sciences and Biotechnologies, Sapienza University of Rome Polo Pontino, Latina, Italy; ^5^IRCCS Neuromed, Pozzilli, Italy; ^6^Institute for Diagnosis and Care “Hermitage Capodimonte,” Naples, Italy

**Keywords:** migraine, headache, pain processing, pain measurement, pain threshold, pain intensity perception, temporal summation

## Abstract

**Background:** It is well-known that both inter- and intra-individual differences exist in the perception of pain; this is especially true in migraine, an elusive pain disorder of the head. Although electrophysiology and neuroimaging techniques have greatly contributed to a better understanding of the mechanisms involved in migraine during recent decades, the exact characteristics of pain threshold and pain intensity perception remain to be determined, and continue to be a matter of debate.

**Objective:** The aim of this review is to provide a comprehensive overview of clinical, electrophysiological, and functional neuroimaging studies investigating changes during various phases of the so-called “migraine cycle” and in different migraine phenotypes, using pain threshold and pain intensity perception assessments.

**Methods:** A systematic search for qualitative studies was conducted using search terms “migraine,” “pain,” “headache,” “temporal summation,” “quantitative sensory testing,” and “threshold,” alone and in combination (subject headings and keywords). The literature search was updated using the additional keywords “pain intensity,” and “neuroimaging” to identify full-text papers written in English and published in peer-reviewed journals, using PubMed and Google Scholar databases. In addition, we manually searched the reference lists of all research articles and review articles.

**Conclusion:** Consistent data indicate that pain threshold is lower during the ictal phase than during the interictal phase of migraine or healthy controls in response to pressure, cold and heat stimuli. There is evidence for preictal sub-allodynia, whereas interictal results are conflicting due to either reduced or no observed difference in pain threshold. On the other hand, despite methodological limitations, converging observations support the concept that migraine attacks may be characterized by an increased pain intensity perception, which normalizes between episodes. Nevertheless, future studies are required to longitudinally evaluate a large group of patients before and after pharmacological and non-pharmacological interventions to investigate phases of the migraine cycle, clinical parameters of disease severity and chronic medication usage.

## Introduction

Migraine is one of the most prevalent neurological disorder and consistently ranks fifth to eighth among the top causes of disability, worldwide (1). Ictal migraine manifestations are characterized by episodes of unilateral moderate or severe throbbing and pulsating headache, frequently associated with nausea, vomiting, photophobia, phonophobia, and cutaneous allodynia (CA) (2, 3), defined as “pain due to a stimulus that does not normally provoke pain” (4). Current consensus is that migraine is an episodic, recurrent, genetically determined dysfunction of brain excitability that leads to the activation and sensitization of the trigeminovascular pain pathway. However, the neural components and mechanisms involved in the generation and recurrence of migraine headaches are still far from being understood.

Nevertheless, during recent decades, electrophysiology and neuroimaging techniques have greatly contributed to the understanding of the mechanisms involved in this elusive painful disorder. Several neurochemical, functional and microstructural alterations across various subcortical-cortical cerebral areas have been described thus far (5–7). However, despite enormous progress in the field, one of the biggest challenges of researchers worldwide remains the identification of the brain circuitry sub-serving and encoding the clinical perception of pain in migraine.

It is well-known that the perception of clinical pain *per se* appears to be vary greatly from person to person in the general population. However, there also exists intra-individual differences. Indeed, it is well-known that several factors, such as spontaneous neuronal fluctuations, attention, expectation of pain, cognitive and emotional states, sleep habits and stress, may influence pain perception (8).

Studies of pain perception in humans require an external stimulus that must be applied to recreate the experience of pain. Once produced, this artificial experience of pain can be evaluated by verbal, behavioral, and physiological measures (9). The single-point measure of “pain threshold” (PT)—defined as the minimum intensity of a stimulus that is perceived as painful (4)—is often used to easily assess subjective pain sensitivity. In an experimental setting, the subject indicates the moment in which a stimulus is perceived as painful, corresponding to a detailed definition of the stimulus itself (e.g., degrees centigrade for heat and cold stimuli, milliamperes for electrical stimuli, grams for pressure with Von Frey filament, etc.).

In contrast to the well-established response related to PT evaluation, the assessment of perceived pain intensity (PPI) consists of delivering a series of discrete supra-threshold stimuli of varying intensity in random order (9). This method assumes that subjects can judge the magnitude of a pain-inducing stimulus, primarily using a rating scale (e.g., Numerical Rating Scale from 0 to 10 or 100).

To better understand migraine-related changes in pain processing, several authors have evaluated pain perception during different phases of the migraine cycle using pressure, electrical, and thermal (cold or heat) stimuli, single or repetitive pulses of varying frequencies delivered over cephalic, cervical, or extra-cephalic areas.

The purpose of this review is to provide a comprehensive overview of the findings of clinical, electrophysiological and functional neuroimaging studies aimed to investigate changes in pain perception of migraine patients during the various phases of the so-called migraine cycle, using PT and PPI assessments.

## Review criteria

We initially searched the PubMed database to identify articles published up to December 2016. The search terms used were “migraine,” “pain,” “headache,” “temporal summation,” “quantitative sensory testing,” and “threshold,” alone and in combination. The literature search was updated using the additional keywords “pain intensity,” and “neuroimaging” to identify full-text papers written in English and published in peer-reviewed journals, using the PubMed and Google Scholar databases. In addition, we manually searched the reference lists of all research articles and review articles.

Data were presented with the criteria of following the various phases of the migraine cycle, and the progression of migraine (see Tables 1–3 for further information).

**Table 1:** Pain threshold findings from experimental studies during noxious stimulation in migraine patients.

**References**	**Subjects**	**Migraine phase**	**Stimulus**	**Area of stimulation**	**Main findings in migraine**
Sand et al. (10)	Episodic migraine patients (*N* = 41) and HCs (*N* = 28)	Preictal	Heat and cold	Trigeminal and extratrigeminal	Subclinical reduced PT compared to baseline
Burstein et al. (2)	Episodic migraine patients with (*N* = 33) and without (*N* = 9) CA	Ictal and interictal	Heat, cold and pressure	Trigeminal and extratrigeminal	Reduced PT in facial skin (79%) and forearm (67%). The authors found a positive correlation between frequency of headache and extension of low PT area
Burstein et al. (11)	One episodic migraine patient with CA	Ictal and interictal	Heat, cold, mechanical and pressure	Trigeminal and extratrigeminal	Progressive lowering of PT in the facial skin and of ipsilateral forearm skin during the pain acme
Uglem et al. (12)	Episodic migraine patients (*N* = 49) and HCs (*N* = 31)	Ictal and interictal	Heat and cold	Trigeminal and extratrigeminal	Reduced cold PT in the ictal phase compared to the interictal, preictal and postictal phases. No differences in heat PT
De Tommaso et al. (13–16)	Episodic migraine patients (*N* = 10, *N* = 18, *N* = 9, *N* = 18)	Interictal and ictal	CO_2_-laser	Trigeminal and extratrigeminal	Reduced PT during the migraine attack compared with baseline and related with duration of illness
Engstrøm et al. (17)	Episodic migraine patients with and without aura (*N* = 50), and HCs (*N* = 34)	Interictal phase	Heat, cold and pressure	Trigeminal and extratrigeminal	Lower thermal PT in migraine patients compared to HC. Lower PT seems related to increased sleep pressure
Schwedt et al. (18)	Chronic and episodic migraine patients (*N* = 112) and HCs (*N* = 75)	Interictal phase	Heat	Trigeminal and extratrigeminal	Lower heat PT in migraine patients compared to HC
Schoenen et al. (19)	Episodic female migraine patients without aura (*N* = 10) and HCs (*N* = 20)	Interictal phase	Pressure	Trigeminal	Lower forehead PT in migraine patients compared to HC
Sandrini et al. (20)	Episodic migraine patients without aura (*N* = 48) and HCs (*N* = 24)	Interictal phase	Electrical	Trigeminal (cornea)	Lower corneal pain thresholds in migraine patients compared to HC; the lowest values were observed on the symptomatic side of unilateral migraine patients
Fernández-de-Las-Peñas et al. (21)	Episodic migraine patients with unilateral pain (*N* = 25) and HCs (*N* = 25)	Interictal phase	Pressure	Trigeminal (temporalis and trapezius muscles)	Lower pressure PT on the symptomatic side as compared with the non-symptomatic side and to either side in controls; no significant differences between the non-symptomatic side and HC
Fernández-de-las-Peñas et al. (22)	Episodic migraine patients (*N* = 15) and HCs (*N* = 15)	Interictal phase	Pressure	Trigeminal (nine points of the temporalis muscle)	Lower pressure PT than HC. Lower PPT in the center compared with the posterior part of the muscle
Fernández-de-las-Peñas et al. (23)	Episodic migraine patients (*N* = 20) and HCs (*N* = 20)	Interictal phase	Pressure	Trapezius muscle (eleven points of the trapezius muscle)	Lower pressure PT levels in the trapezius muscle region compared with HC
Zappaterra et al. (24)	Chronic (*N* = 44) and episodic (*N* = 21) migraine patients and HCs (*N* = 26)	Interictal phase	Pressure	Trigeminal (temple, cheekbone, and cervical areas)	Lower pressure PT in chronic migraine patients and medications overuse; no difference of mechanical PT in episodic migraine patients
Grossi et al. (25)	Chronic (*N* = 14) and episodic (*N* = 15) migraine patients and HCs (*N* = 15)	Interictal phase	Pressure	Trigeminal (frontalis, temporalis, masseter) and extratrigeminal (trapezius and sternocleidomastoid)	Decreased PPT in women with migraine relative to HC; no significant PPT values differences between episodic migraine patients and chronic migraine patients
Schwedt et al. (26)	Chronic (*N* = 20) and episodic (*N* = 20) migraine patients and HCs (*N* = 20)	Interictal phase	Heat, cold and pressure	Trigeminal (forehead) and extratrigeminal (ventral forearms)	Lower thermal PT in both chronic and episodic migraine patients compared to HC; no difference in the mechanical PT
Florencio et al. (27)	Episodic migraine patients (*N* = 30) and HCs (*N* = 30)	Interictal phase	Pressure	Trigeminal (sternocleidomastoid, suboccipital, trapezius, scalene)	Lower pressure PT in all muscles compared with controls
Palacios-Ceña et al. (28)	Chronic (*N* = 52) and episodic (*N* = 51) female migraine patients and HCs (*N* = 52)	Interictal phase	Pressure	Trigeminal (temporalis muscle), extratrigeminal (second metacarpal, tibialis anterior muscle)	Lower pressure PT over trigeminal and extra-trigeminal points
Bovim (29)	Episodic migraine patients with and without aura (*N* = 26) and HCs (*N* = 20)	Interictal phase	Pressure	Trigeminal twenty-two specified points (11 on each side of the head)	No significant side-to-side differences in the pressure PT in patients with strict unilateral migraine, between migraine patients and HC
Göbel et al. (30)	Episodic migraine patients with migraine without aura (*N* = 23) and HCs (*N* = 29)	Interictal phase	Pressure	Trigeminal	No significant differences in pain sensitivity of pericranial musculature
Bishop et al. (31)	Episodic migraine patients with migraine without aura (*N* = 27) and HCs (*N* = 27)	Interictal phase	Cold	Extratrigeminal (non-dominant hand)	No significant difference in the cold pressor test
Weissman-Fogel et al. (32)	Episodic migraine patients with and without aura (*N* = 34) and HCs (*N* = 28)	Interictal phase	Heat and electric	Trigeminal (periorbital area) and extratrigeminal (forearm)	No significant differences in mean heat, mechanical, and electrical PT between migraineurs and controls
Katsarava et al. (33)	Episodic migraine patients (*N* = 17) and HCs (*N* = 15)	Ictal and interictal phase	Electric (nociceptive blink reflex)	Trigeminal	
Ayzenberg et al. (34)	Chronic (*N* = 29) and episodic (*N* = 16) migraine patients and HCs (*N* = 15)		Electric (nociceptive blink reflex)	Trigeminal (periorbital area) and extratrigeminal (hand)	No significant differences between HC and patients with episodic migraine and depression without headache
Buchgreitz et al. (35)	Episodic migraine patients (*N* = 60) and HCs (*N* = 294)	Interictal phase	Pressure	Trigeminal (dorsum of the second finger) and extratrigeminal (anterior part of the temporal muscle)	No significance differences in pressure PT
Coppola et al. (36)	Episodic migraine patients without aura (*N* = 14) and HCs (*N* = 15)	Interictal phase	Electric (nociceptive blink reflex)	Trigeminal (supraorbital area) and extratrigeminal (index finger)	No significant differences between migraine patients and HC for pain threshold
Gierse-Plogmeier et al. (37)	Episodic female migraine patients with (*N* = 10) and without (*N* = 10) aura and HCs (*N* = 20)	Interictal phase	Electrical	Trigeminal (masseter region) and extratrigeminal (tibial region)	No differences respect to pure sensory and pain electric thresholds neither for the peripheral nor for the trigeminal stimulation
Perrotta et al. (38)	Patients with MOH (*N* = 31), episodic (*N* = 28) migraine patients and HCs (*N* = 23)	Interictal phase	Cold	Extratrigeminal	Significantly reduced mean electric PT in the MOH patients (both bWT and aWT) compared with HC; and in the MOH patients bWT compared with both the MOH patients aWT and the EM patients.
Teepker et al. (39)	Episodic female migraine patients with and without aura (*N* = 26), HCs (*N* = 13)	Interictal phase	Heat, cold, pressure and electrical	Extratrigeminal	No significant differences in PT; no relationship between PT and oral contraceptives assumption
Zohsel et al. (40)	Children affected by migraine with and without aura (*N* = 25), HCs (*N* = 28)	Interictal phase	Heat, pressure	Trigeminal (upper cheek) and extratrigeminal (thenar eminence)	Heat PT was not significantly different between the two groups. However, the child migraineurs showed significantly lower mechanical PT
De Tommaso et al. (41)	Children affected by migraine without aura (*N* = 34) and HCs (*N* = 17)	Interictal phase	Laser	Trigeminal (supraorbital area) and extratrigeminal (hand)	Laser PT was significantly reduced in child migraineurs compared to HC at the hand level, but this difference was not significant at the trigeminal site
Ferracini et al. (42)	Children affected by migraine without aura (*N* = 50) and HCs (*N* = 50)	Interictal phase	Pressure	Trigeminal and extratrigeminal	The pressure PT did not differ between children with migraine and children without headache;
Anttila et al. (43)	Children affected by migraine without aura (*N* = 59) and HCs (*N* = 59)	Interictal phase	Pressure	Trigeminal and extratrigeminal (seven pericranial and neck-shoulder tender points)	The mean pressure PT did not differ among the groups
Metsahonkala et al. (44)	Children affected by migraine without aura (*N* = 48) and HCs (*N* = 59)	Interictal phase	Pressure	Trigeminal and extratrigeminal (seven cephalic and three extracephalic points)	The mean pressure PT did not differ among the groups of the study
Uthaikhup et al. (45)	Elders affected by migraine without aura (*N* = 26) and HCs (*N* = 44)	Interictal phase	Heat, cold, and pressure	Trigeminal (forehead and upper neck) and extratrigeminal (tibialis anterior)	No significant differences between the headache groups and HC in pressure and cold PT
Cooke et al. (46)	Chronic migraine female patients (*N* = 15) and HCs (*N* = 15)	Interictal phase	Pressure	Trigeminal and extratrigeminal	The pressure PT was significantly lower in migraine patients than in HC subjects for the 1st and 2nd division of the trigeminal nerve
Kitaj and Klink (47)	Episodic migraine patients (*N* = 40) and patients with transformed migraine (*N* = 41)	Interictal phase	Heat, cold, mechanical, and pressure	Trigeminal (forehead, maxillae) and extratrigeminal (C4 dermatome, and forearms)	Significantly lower pain thresholds in patients with transformed migraine compared to episodic migraine patients
De Tommaso et al. (48)	Chronic migraine patients (*N* = 25), migraine patients without aura (*N* = 15) and HCs (*N* = 15)	Interictal phase	CO2-laser thermalstimulation	Trigeminal (face) and extratrigeminal (hand)	No significant differences in PT at both the hand and the face between the three groups
De Tommaso et al. (49)	Chronic migraine patients (*N* = 16), migraine patients with aura (*N* = 10) and HCs (*N* = 15)	Interictal phase	CO_2_-laser pulses	Trigeminal	No significant differences in the PT; prevalent activation of the rostral portion of the ACC in patients with chronic migraine
De Tommaso et al. (50)	Chronic migraine patients (*N* = 16) and HCs (*N* = 16)	Interictal phase	CO_2_-laser	Extratrigeminal	No significant differences in the PT; different modulation of bilateral parietal cortical areas and superior frontal and cingulate girus induced by different virtual reality in patients with CM compared to HC
Ferraro et al. (51)	Patients with MOH (*N* = 14) and HC (*N* = 14)	Interictal phase	Laser	Trigeminal and extratrigeminal	No significant differences in the PT; deficient habituation of the vertex N2/P2 complex partly restored after successful treatment of MOH

*CA, cutaneous allodynia; HCs, healthy controls; MOH, medication overuse headache; PT, pain threshold*.

**Table 2:** Perceived pain intensity findings from experimental studies during heat, cold, pressure, or electrical noxious stimulation in migraine patients.

**References**	**Subjects**	**Migraine phase**	**Stimulus/i**	**Area of stimulation**	**Main findings in migraine**
Burstein et al. (2)	Episodic migraine patients with (*N* = 33) and without (*N* = 9) CA	Ictal and interictal	Heat and cold	Trigeminal and extratrigeminal	Higher PPI values during ictal, compared to interictal, period
Burstein et al. (11)	One episodic migraine patient with CA	Ictal and interictal	Heat, cold and pressure	Trigeminal and extratrigeminal	Higher PPI values during ictal, compared to interictal, period
Uglem et al. (12)	Episodic migraine patients (*N* = 41), HCs (*N* = 31)	Pre-ictal, ictal, post-ictal, and interictal	Heat and cold	Trigeminal	Interictal linear increase of PPI approaching to the next migraine attack, followed by an abrupt pre-ictal PPI decrease, and a subsequent ictal PPI increase.
Weissman-Fogel (32)	Migraine patients (*N* = 34) and HCs (*N* = 28)	Interictal	Heat, electrical and mechanical	Trigeminal and extratrigeminal	Heat PPI values did not differ from those of HCs; whereas electrical and mechanical PPI to phasic stimulations increased in migraine patients when compared with HCs
Gierse-Plogmeier et al. (37)	Migraine with (*N* = 10) and without (*N* = 10) aura and HCs (*N* = 20)	Interictal	Electrical and cold	Trigeminal	Increased interictal PPI with a positive correlation between the PPI and migraine frequency

*HCs, healthy controls; PPI,pain perception intensity*.

**Table 3:** Perceived pain intensity findings from experimental studies using pain-related evoked potentials and neuroimaging during noxious stimulation in migraine patients.

**References**	**Subjects**	**Migraine phase**	**Stimulus/i**	**Area of stimulation**	**Main findings in migraine**
Moulton et al. (52)	Episodic migraine patients with and without aura experiencing CA (*N* = 11), HCs (*N* = 11)	Ictal and interictal	Pain PT + 1°C Heat	Trigeminal and extratrigeminal	No significant between ictal and interictal PPIs
Stankewitz et al. (53)	Episodic migraine patients (*N* = 20), HCs (*N* = 20)	Pre-ictal, ictal, and interictal	Gaseous ammonia	Trigeminal	No significant between groups PPIs differences
Stankewitz et al. (54)	Episodic migraine patients (*N* = 20), HCs (*N* = 20)	Ictal and interictal	Gaseous ammonia	Trigeminal	No significant between groups PPIs differences
Schulte and May (55)	One episodic migraine with aura patient	Pre-ictal, ictal, and interictal	Gaseous ammonia	Trigeminal	Increased PPI during the ictal phase compared with pre-ictal and interictal phases
De Tommaso et al. (56)	Migraine without aura patients (*N* = 8) and HCs (*N* = 10)	Interictal	CO_2_ laser stimulation	Trigeminal	No significant baseline between groups PPI differences. A greater PPI in response to laser stimulation has been demonstrated in migraine patients compared to HC after capsaicin application
De Tommaso et al. (57)	Migraine without aura patients (*N* = 10) and HCs (*N* = 7)	Interictal	CO_2_ laser stimulation	Trigeminal and extratrigeminal	No significant between groups PPI differences, during warning stimulus
De Tommaso et al. (58)	Migraine without aura patients (*N* = 14) and HCs (*N* = 10)	Interictal	CO_2_ laser stimulation	Trigeminal	PPI in response to laser stimulations non-significantly declined across repetitions in migraine patients, while was significantly reduced in HC
De Tommaso et al. (59)	Migraine without aura patients (*N* = 12) and HCs (*N* = 10)	Interictal	CO_2_ laser stimulation	Trigeminal and extratrigeminal	No significant baseline between groups PPI differences. A greater PPI in response to laser stimulation has been demonstrated in migraine patients compared to HC after capsaicin application
De Tommaso et al. (60)	Migraine without aura patients (*N* = 8) and HCs (*N* = 8)	Interictal	CO_2_ laser stimulation	Trigeminal and extratrigeminal	No significant between groups PPI differences, during discrimination and arithmetic tasks
Moulton et al. (61)	Episodic migraine patients experiencing CA (*N* = 12) and HC (*N* = 12)	Interictal	41°C innocuous heat stimulus and PT+1°C	Trigeminal and extratrigeminal	No significant between groups PPI differences
De Tommaso et al. (62)	Migraine without aura female patients during the pre-menstrual and late luteal phases (*N* = 9) and HCs (*N* = 15)	Interictal	CO_2_ laser stimulation	Trigeminal and extratrigeminal	No significant between groups PPI differences, during the pre-menstrual phase
De Tommaso et al. (63)	Migraine without aura patients (*N* = 24) and HCs (*N* = 22)	Interictal	CO_2_ laser stimulation	Trigeminal and extratrigeminal	No significant between groups PPI differences, during affective picture viewing
De Tommaso et al. (64)	Migraine without aura patients (*N* = 13) and HCs (*N* = 12)	Interictal	CO_2_ laser stimulation	Trigeminal and extratrigeminal	No significant between groups PPI differences, after excitability enhancers repetitive transcranial magnetic stimulation
Di Clemente et al. (65)	Episodic migraine patients with and without aura (*N* = 13) and HCs (*N* = 15)	Interictal	Nd-YAP laser stimulation	Trigeminal and extratrigeminal	No significant between groups PPI differences
Ferraro et al. (51)	Medication overuse headache patients (*N* = 14) and HCs (*N* = 14)	Interictal	CO_2_ laser stimulation	Trigeminal and extratrigeminal	They found that PPI to laser stimuli significantly showed delayed habituation in patients, while after treatment, non-responders (true CM patients) showed lack of habituation in comparison to both HC and responders
Russo et al. (66)	Migraine patients (*N* = 16) and HCs (*N* = 16)	Interictal	41°C innocuous, 51°C moderate and 53°C high-noxious heat stimuli	Trigeminal	No significant between groups PPI differences
Schwedt et al. (67)	Episodic migraine patients with and without aura (*N* = 24) and HCs (*N* = 27)	Interictal	Suprathreshold heat stimulus	Trigeminal and extratrigeminal	No significant between groups PPI differences
Beese et al. (68)	Migraine patients (*N* = 22) and HCs (*N* = 22)	Interictal	51°C heat stimulus	Trigeminal and extratrigeminal	No significant between groups PPI differences
De Tommaso et al. (69)	Migraine without aura patients (*N* = 31) and HCs (*N* = 19)	Interictal	CO_2_ laser stimulation	Extratrigeminal	Higher baseline laser PPI in migraine than HC
Mathur et al. (70)	Migraine patients (*N* = 14) and HCs (*N* = 14)	Interictal	Thermal	Extratrigeminal	Pain ratings were higher for patients than HC in response to heat stimuli of moderate intensity
Vecchio et al. (71)	Migraine without aura patients (*N* = 32) and HCs (*N* = 16)	Interictal	CO_2_ laser stimulation	Trigeminal and extratrigeminal	Higher baseline laser PPI in migraine than HC. PPI values did not change after excitability enhancers direct current stimulation
Russo et al. (72)	Migraine patients not experiencing ictal CA (*N* = 20), migraine patients experiencing ictal CA (*N* = 20) and HCs (*N* = 20)	Interictal	41°C innocuous, 51°C moderate and 53°C high-noxious heat stimuli	Trigeminal	Migraine patients with and without CA showed similar PPI values in comparison with HCs
Russo et al. (73)	Migraine patients not experiencing CA (*N* = 34), migraine patients experiencing CA (*N* = 30), migraine with aura (*N* = 30) and HCs (*N* = 30)	Interictal	41°C innocuous, 51°C moderate and 53°C high-noxious heat stimuli	Trigeminal	Migraine patients with and without CA showed PPI values comparable to that of migraine with aura patients and HCs

*HCs, healthy controls; PPI, pain perception intensity*.

## Data overview

### Ictal phase of episodic migraine

Few studies were dedicated to the assessment of PT in migraine patients during a migraine headache (ictal phase).

#### Pain threshold

Burstein and colleagues repeatedly measured PT both in the absence and during the course of moderate-to-severe migraine attacks in the same patients using bilateral heat, cold and pressure stimuli applied to the cephalic (forehead) and extra-cephalic (forearm) regions (2). In this study, 79% of patients showed reduced PT by at least one standard deviation from the baseline interictal control threshold on the facial skin ipsilateral to migraine pain, as assessed by one or more modalities. This was considered a clinical manifestation of CA. Frequently, CA was not restricted to the referred migrainous pain area, but spread contralaterally, and even to the ipsi- and contra-lateral forearms. Notably, patients' PT were compared among themselves (interictal vs. ictal) rather than to healthy controls (HC). Interestingly, non-allodynic patients were younger, with shorter disease duration, and less frequently reported aura symptoms compared to allodynic patients.

In a 42 year old male affected by migraine with aura (MwA), Burstein and colleagues (11) further investigated the temporal and spatial development of CA. In the first phase of a migraine attack, a reduced PT for mechanical and cold stimuli was found within the referred pain area. Two hours from the onset of migraine attack, further lowering of mechanical PT on the ipsilateral forehead and lowering of pressure and cold PT on the contralateral forehead and ipsilateral forearm were observed. Finally, during the acme of the migraine attack (4 h from the onset), heat PT was even further decreased in both the ipsilateral and contralateral forehead and in the ipsilateral forearm (11). Other observations based on the longitudinal study of a group of patients (10, 12) supported that PT is consistently lower during the ictal phase compared to the interictal phase. Focusing on a more recent study performed in a large cohort of participants, authors observed decreased cold PT of the forehead during the ictal phase compared to the interictal phase. However, no differences in the heat PT of the hand or forehead was observed between ictal and interictal phases (12). These contrasting data (2, 11) could be related to the time-point within the development of the migraine attack when testing took place. However, PT was not altered by controlling for aura or headache side.

#### Pain perception intensity as judged by pain-related evoked potentials

In migraine, the PPI assessment during continuous suprathreshold heat pain stimulation on the temporal areas showed significantly higher values during the ictal period than during interictal period (2, 11). This was not altered by controlling for aura or the headache side. Notably, an alternative experimental approach to study pain processing is by delivering repetitive laser stimulations, and rating PPI while recording cortical laser evoked potentials (LEPs). In electrophysiological studies using CO_2_ laser stimulations, lowered basic PT and resulting increased cortical amplitudes during spontaneous or experimentally induced migraine attacks have been confirmed in the bilateral cephalic (supraorbital) and extra-cephalic areas (hand) ipsilateral to headache side compared to the interictal phase (13–16). Interestingly, administration of either almotriptan or lysine-acetylsalicylate could not increase PT to laser stimuli in migraine patients for up to 2 h after intake, though treatment provided relief from headache (16).

#### Pain perception intensity as judged by functional neuroimaging

Recently, increased trigeminal PPI using trigeminal nociceptive stimuli (gaseous ammonia) has been demonstrated during the ictal phase compared with preictal and interictal phases in one migraine patient scanned daily for 30 days (55). In this patient, the hypothalamus was significantly more active immediately before the headache phase, when it also showed the greatest functional coupling with the spinal trigeminal nuclei. On the other hand, during the ictal state the hypothalamus was functionally coupled with the dorsal rostral pons. However, in a similar (though retrospective) study from the same research group, no differences in PPI were observed using olfactory stimulations in episodic migraine patients during the interictal, preictal, or ictal phases, or when compared with HC (53).

Overall, consistent data indicate that PT is lower during the ictal phase than both the interictal phase of migraine and in HC in response to pressure, cold, and heat stimuli applied over the cephalic (forehead) region on the usual headache side. The involvement of extracephalic territories (forearm or hand) ipsilateral and/or contralateral to the headache side depends mainly on the time that has passed since the beginning of the attack. PPI during continuous suprathreshold heat pain stimulation at the cephalic and extracephalic territories was significantly higher in patients in the ictal phase, than in those in the interictal phase or in HC. These changes in PT and PPI during the ictal phase are accompanied by a heightened cortical response and reorganization of brain areas devoted to nociception/antinociception.

### Peri-ictal phases of episodic migraine

Although the abnormalities in the pain perception are more pronounced during migraine attacks, subtle changes may be present before and after the headache phase (i.e., during the so-called preictal and postictal phases). The preictal phase occurs a couple of hours to days before the onset of a migraine attack, and is manifested by various symptoms such as fatigue, difficulty in concentrating, neck stiffness, increased sensitivity to light and sound, nausea, blurred vision, yawning, and pallor. The postictal phase usually lasts 24 h, and is frequently associated with mood changes, muscle weakness, physical exhaustion, and reduced appetite (74).

#### Pain threshold

Until now, few studies have directly or indirectly focused on PT in the preictal and postictal phases of the migraine cycle. Sand and colleagues (10) performed a blinded paired longitudinal investigation of 11 patients (about 24 h before the headache), and performed within-subject comparisons between the preictal and interictal phase. The authors found that preictally, heat PTs were slightly lower in the forehead, neck and hand, while cold PTs were lower in the neck and hand. Moreover, in preictal phase, significant lower cold (but not heat) PTs were detected on the symptomatic forehead side when compared with the asymptomatic side. Although the researchers did not check for possible differences in PT between preictal migraine patients and HC, only marginal differences between preictal data and HC can be observed in the raw data presented (10). The mild decrease in PT (remaining within the normal range) was considered suggestive of preictal heat sub-allodynia (12, 29). However, despite these encouraging results, another small study performed by the same research group did not find significant differences in pressure and thermal PT between interictal, preictal, and postictal migraine patients (17). These negative results were recently confirmed in a group of migraine patients comparing both interictal-ictal and interictal-postictal paired measurements (12). Nonetheless, they emphasized that both heat and cold PT values were lower in the ictal compared to the interictal phase, arguing that an interictal-preictal-ictal gradient could exist. Indeed, when interictal and preictal patients were pooled and analyzed over a 15-day range, it is possible that preictal thermal sub-allodynia manifests closer to the attack in many episodic migraine patients (12). The latter data seem to be in line with the previous observations of a positive correlation between heat PT (at the forehead and at the forearm) and the number of hours until the next migraine attack (18). This unusual pattern suggests that neural events affect pain processing the day before the migraine attack (12). However, study limitations included PT evaluation regardless of the side on which the patient most commonly experienced headaches, and the inclusion of patients taking acute medication, which may have biased analyses.

Moreover, in a study evaluating the relationship between PT and sleep quality in migraine, slow-wave sleep was negatively correlated with pressure PT, and slow bursts were negatively correlated with thermal PT, suggesting that sleep abnormalities might precipitate attacks and induce hyperalgesia (17).

#### Pain perception intensity as judged by quantitative sensory testing

The fluctuations in pain processing of migraine patients depending on the time from attack onset has been further confirmed by Uglem et al. (12). They assessed subjective PPI and showed a linear increase of PPI during the interictal period approaching the next migraine attack, followed by an abrupt preictal PPI decrease, and a subsequent ictal PPI increase.

Overall, during the preictal phase, at a time when premonitory symptoms may occur, patients tend to show only mildly significant lowered PT to heat and cold stimuli in the forehead, neck, and hand, mainly on the symptomatic side, compared to their own interictal phase or to HC. This condition was considered to be suggestive of preictal sub-allodynia and could be considered an early manifestation of (or a predisposing factor for) the development of the next attack. Some studies suggest that migraine patients experience fluctuations in pain processing depending on the distance from the attack, since PT decreases preictally approaching the next attack, while PT greatly decreased and PPI increased ictally.

### Interictal phase of episodic migraine

#### Pain threshold

Several studies using different paradigms have investigated thermal, mechanical, or electrical PT in migraine patients in the interictal phase and compared them with HC. However, these studies have generated conflicting results, showing significant differences in PT between migraine patients and HC (17–28) or no differences (10, 29–39, 68, 69, 75). Among the work in which a significantly lower PT has been demonstrated in migraine patients, PT reduction was observed in cephalic (17–22, 24–26, 28), cervical (21, 23, 25, 27), and extracephalic (18, 21, 25, 26, 28, 69) regions, in particular using mechanical painful stimuli (19, 21–25, 27, 28), to a lesser extent in thermal (18, 26, 69, 76), and in one case electric (20) stimuli.

Fernández-de-las-Peñas et al. showed that pressure PTs in patients with strictly unilateral migraine was significantly lower in the cervical (upper trapezius muscle), but not cephalic areas of the symptomatic side compared to the non-symptomatic side or HC (21, 23). Further, this effect was more evident in females than in males (21). However, another group of researchers failed to find a correlation between headache laterality and pressure PT values in a group of women with migraine tested interictally (25). In a large cohort of mixed episodic and chronic migraine (CM) patients, Schwedt et al. (18) failed to confirm their previous findings of a negative correlation between heat (but not cold or pressure) PT and CA symptom scores, as assessed at the time of testing or recalled during headache (26). This was despite the observation of increased (but within normal range) self-reported CA scores.

Taken together, these studies demonstrate great variability in results, likely due to a highly heterogeneous subject samples. For instance, studies included patients on prophylactic medication, or in some sub-analyses, intermixed (e.g., episodic and chronic) migraine patients (21, 27, 77). Nonetheless, a major source of inconclusiveness in studies is in the lack of precise information regarding the time-point during the migraine cycle in which patients were tested, as authors did not control for proximity to the next attack (18, 21–24, 26, 27, 77).

#### Pain perception intensity as judged by pain-related evoked potentials

Using heat LEPs, the majority of studies did not report differences in laser PPI between migraine patients during the pain-free phase and HC (57, 59, 65), after visual warning stimulus (57), during discrimination and arithmetic tasks (60), during the pre-menstrual phase (62), during affective picture viewing (63), or after administration of excitability enhancers such as repetitive transcranial magnetic (64) or direct current (71) stimulations. Nonetheless, some exceptions exist which found higher baseline laser PPI in migraine patients compared to HC (69, 71).

The absence of PPI differences between migraine patients during the pain-free phase and HC has been supported by robust behavioral data collected during event-related neuroimaging studies using noxious heat stimuli, although based on small heterogeneous migraine patient populations (52–54, 61, 66, 67, 72). These findings have been confirmed in a subgroups of episodic migraine patients with or without CA compared to HC (72), when stimuli were applied both over cephalic (52–54, 61, 66, 67, 72) and extra-cephalic regions (52, 67). Moreover, PPI ratings did not differ between pain-free patients and HC using both variable heat PT+1°C stimulus intensity (52, 61, 67) or fixed moderate-to-high painful heat stimuli (66, 72), with one notable exception where pain ratings were higher for migraine patients in response to moderate heat stimuli (70).

A greater PPI in response to laser stimulation has been demonstrated in migraine patients compared to HC after capsaicin application, with less inhibition of laser evoked potential (LEP) amplitudes (56, 59). PPI and LEP amplitudes in response to laser stimulations non-significantly declined across repetitions in migraine patients between attacks, while both were significantly reduced in HC- a behavioral phenomenon ascribed to a sensitization mechanism rather than habituation (58). Similarly, some authors found increased PPI in migraine patients between attacks compared to HC by delivering repetitive trains of mechanical (32) and electrical (32, 37, 38) pain stimuli, an experimental model of sensitization also knows as temporal summation (or “wind-up”). Patients with increased temporal summation had increased frequency of attacks per month and tended to be closer to their last attack than patients without increased temporal summation (32, 37). The authors ascribed this phenomenon to sub-clinical activation-dependent plasticity of pain pathways, reflecting subtle increased membrane excitability that may be described clinically as a sub-allodynia state.

#### Pain perception intensity as judged by functional neuroimaging

Neuroimaging studies in patients scanned during the migraine attacks either documented significant greater PPI than during the interictal phase (55), or no differences (53–54).

Compared to HC, migraine patients are characterized by an increased blood oxygenation level dependent (BOLD)–response in brain areas involved in nociception/antinociception and neurocognitive aspects of pain processing (i.e., insula, middle cingulate and anterior cingulate cortices, secondary somatosensory cortex, amygdala, cerebellum, caudate nuclei, and motor and pre-motor areas, temporal pole, lentiform nuclei, posterior thalamus, fusiform gyrus, subthalamic nucleus, pre- and post-central gyrus, hippocampus and parahippocampal gyrus, and dorsolateral prefrontal cortex) (52–54, 67, 70). Further, they exhibit reduced BOLD activation within the brainstem (trigeminal nucleus caudalis, nucleus cuneiformis) (53, 61), despite experiencing normal heat PPI in cephalic and extra-cephalic areas.

Interestingly, spinal trigeminal nucleus activation appears to fluctuate during the migraine cycle, being slightly greater in the preictal state compared to HC and significantly enhanced compared to interictal scans. and decreased during an acute attack compared to both HC and preictal migraine patients (53).

Russo and colleagues used fixed innocuous (41°C), moderate noxious (51°C), and severe noxious (53°C) heat stimuli over the innervation territory of the maxillary division of the trigeminal nerve delivered on the more affected head side in episodic migraine patients between attacks during MRI scans (66, 72). The authors did not observe differences in cerebral BOLD signals during the innocuous (41°C) stimulus sessions compared to HC, or in subgroups with or without CA. However, while PPI was not significantly different between patients and HC, an overall greater activation to a moderate painful stimulus was observed in the perigenual area of the anterior cingulate cortex (66), especially in patients experiencing CA (72). This was associated with further involvement of the left anterior pons within the brainstem (66) and the left middle frontal gyrus (72). During the severe painful stimulus, an overall increased activation of the secondary somatosensory cortex was observed bilaterally in migraine patients (66) and in the subgroups with and (even more so) without CA (72).

There were no statistically significant correlations between PPI at any level of experimental stimuli nor BOLD-fMRI signal changes in migraine patients with or without experience of CA (72). Furthermore, there was no significant correlation between pain-induced BOLD signal changes and depression or state-trait anxiety scores (67). Nonetheless, when clinical migraine features were regressed with pain-related BOLD signals, the time to next attack was positively related to activation strength within the trigeminal nuclei (53), intensity of headache was negatively related to middle prefrontal cortex and posterior cingulate cortex and positively to bilateral insula activation (70), attack frequency was related to activation strength within several brain areas (i.e., middle cingulate, insula, fusiform gyrus, hippocampus, dorsolateral prefrontal cortex, precentral gyrus) (52, 67, 70), and years with migraine was positively related to activation strength within the fusiform gyrus (67) and negatively related to that in the superior temporal gyrus (70).

More recently, Russo and colleagues (73) used trigeminal heat stimulation (THS) at three different predefined intensities (41, 51, and 53°C), and explored PPI in three drug-naïve patient groups characterized by homogeneous migraine phenotypes such as migraine without aura (MwoA) without CA (MwoA CA−), MwoA with ictal CA (MwoA CA+), and MwA without CA (MwA CA−). When compared to HC, the authors found no significant differences in PPI in any experimentally induced stimuli. Moreover, no significant correlations were found between clinical variables and PPI of the THS at any level of experimental stimulus.

Overall, the results obtained during the interictal period are conflicting, because either reduced or no differences in PT were observed. However, studies were mostly conducted using mechanical sensory testing, making it impossible to determine the contribution of the peripheral muscle (e.g., muscle tenderness) and central sensitization processes. Indeed, pressure PT was unrelated to the presence of overt interictal/ictal CA. Quantitative sensory testing and neuroimaging data support the concept that migraine patients may be characterized by a normal PPI, despite methodological limitations of the studies. Nonetheless, some authors detected increased PPI during concomitant sensitization-inducing stimulations (such as stimulus repetition and capsaicin application) in migraine patients between attacks compared to HC, especially when patients were tested closer to an attack or experienced higher attack frequency. Finally, neuroimaging studies reveal that, despite essentially normal PPI ratings, the migraine brain encodes and behaves differently to subjective PPI compared to HC, especially depending on patients' clinical features.

#### Pain perception in children and elders with migraine

Although migraine in children and the elderly may represent good models to test disease onset and progression, few studies have been dedicated to the assessment of the pain processing in childhood and elderly migraine patients. Nonetheless, research that has been done has also produced contradictory results.

Indeed, both significant differences (40–42), or no differences (43, 44) in PT have been documented in children with migraine during interictal period and HC. Among the research reporting significantly reduced PT in migraine patients, it has been observed in cephalic (40), cervical (42), and extracephalic (40, 41) regions, using mechanical (40, 42) and CO_2_ laser (41) painful stimuli.

In a small cohort of children with a short migraine history, unaltered heat PT and lower mechanical PT were observed following stimulation within the innervation territory of the second trigeminal branch territory (maxilla) as well as the thenar eminence of the non-dominant hand (40).

In a CO_2_ laser evoked potential study, laser PT was significantly lower in children with migraine compared to HC at the hand level; however, no significant differences were observed in the trigeminal field (41). Moreover, in children with headache increased amplitude and lack of habituation of the N2-P2 vertex complex was observed both in the trigeminal and extracephalic areas, which was correlated with acute CA and inter-critical pericranial tenderness (41).

Similar to studies performed in adult migraine patients, some biases affect these observations. For example, the inclusion of patients on prophylactic medication (42) or lack of precise information about the test subject's phase of migraine cycle (40–42) may affect results. Only one study has investigated the effects of pressure, heat, and cold PTs in a group of elderly patients suffering from episodic and CM, mostly experiencing headache on the testing day and taking prophylactic medication (45). The authors did not find significant differences for pressure and cold PT at any somatic location (cephalic, cervical, and extracephalic regions) between the migraine patients and HC. However, the migraine group showed significantly lower heat PT in the upper neck area compared to HC, which was significantly related to the presence of pain in the area, making the PT difference difficult to ascribe to the presence of headache. There were no significant correlations between any of the patients' clinical features and PTs (45).

To the best of our knowledge, there are no studies specifically exploring PPI in children or elders with migraine. Overall, some reports in children with migraine tend to indicate that abnormal noxious information processing could appear early in life, perhaps with a concomitant presence of an altered PT detection system.

#### Pain perception in chronic migraine

A proportion of episodic migraine patients experience an attack frequency equal to or greater than 15 days per month for at least 3 months leading to CM. In clinical practice, the most frequent exogenous factor that leads to headache chronification is medication overuse, diagnosed in up to 80% of patients. The diagnostic criteria of CM are still hotly debated and thus in continuous evolution. Most published papers assessing PT in CM (25, 46, 47, 78) used the criteria of Silberstein-Lipton for transformed migraine (TM) (79) which shows 93% agreement with the International Classification of Headache Disorders 2004 edition (80).

In the assessment of PTs, researchers compared CM patients with HC (24–26, 28, 34, 38, 46, 48–51) and/or with episodic migraine patients (24–26, 28, 34, 38, 47–49, 78). Again, conflicting results were obtained by these studies, since either significantly reduced (24–26, 28, 38, 46), or normal (34, 48–51) PTs were detected in CM patients. Studies that compared CM with episodic migraine patients either documented equal to (25, 26, 28, 48–51) or greater (24, 47, 78) reduction in the former compared to HC. Among the research in which PTs appear to be significantly lower in CM patients, the reduction was observed in cephalic (24–26, 28, 46, 47, 78), cervical (24, 25, 46, 47, 78), and extracephalic (28, 38, 46, 47, 78) regions. This PT reduction was evident using mechanical (24, 25, 28, 46, 47, 78), thermal (26, 47), and electrical (38) painful stimuli.

In a group of CM due to medication overuse (MOH), Perrotta and colleagues (38) found a markedly reduced temporal summation threshold of the spinal noxious flexion reflex, with an increase in reflex area and PPI in MOH patients compared to both HC and episodic migraine patients. Further, they reported a marked improvement in MOH patients after drug withdrawal treatment. Interestingly, activation of the diffuse supraspinal inhibitory controls by means of cold pressor test did not produce any significant effect on either the neurophysiological or psychophysical parameters before detoxification, but not after (38). In a group of mixed episodic and CM patients, Schwedt and colleagues (26) found that patients showed a significant negative correlation between heat PT and number of symptoms of CA. The authors used a questionnaire assessment administered at the time of testing and recalled during headache, and concluded that lower PT during migraine is due to central sensitization phenomena. However, it must be mentioned that in a larger cohort of patients, the same authors failed to confirm their previous findings (18).

Ferraro et al. studied PPI and LEP amplitudes in response to repetitive laser stimulations in CM and MOH (before and after successful treatment) patients as well as HC. They found that PPI and LEP amplitudes to laser stimuli demonstrated significantly delayed habituation in both patient groups, while after treatment, non-responders (true CM patients) showed lack of habituation compared to both HC and MOH patients. While in those MOH patients who showed a treatment response (true MOH patients), authors found normal habituation (51).

In an intermixed group of chronic tension type headache and MOH patients, the duration of the chronic phase was associated with cutaneous PT reduction, while in the MOH only group, mean cutaneous PT values decreased with increasing daily drug intake (24). Finally, some researchers found that in women with CM, the presence of a widespread reduction in PT is negatively associated with the severity of migraine pain but unrelated to the presence of anxiety or depressive symptoms (28). Limitations of such studies can be the inclusion of CM patients on prophylactic medications (25, 46, 47, 78) intermixed with those overusing acute medications (46, 47), and patients with ongoing concomitant chronic pain conditions (47).

Overall, as for the interictal state, PT data obtained from migraine patients ranging from episodic to CM are inconclusive. However, some have documented lower PT in CM patients compared to HC and episodic migraine patients, almost exclusively using mechanical stimuli. Increased PPI ratings with reduced efficiency of supraspinal descending inhibition controls were documented in CM resulting from medication overuse, before and after drug detoxification. Nonetheless, the level of PT seems to be related to the severity of migraine and the daily drug intake level.

## Discussion

Migraine is presently considered a disorder of the brain. Many independent research groups have observed that the brain of migraine patients abnormally processes all sensory information, with the exception of olfaction (81). These functional abnormalities are not constant, but rather exist under continuous fluctuations following various phases of the so-called migraine cycle (6). Recent evidence provided by modern MRI techniques tends to show that reversible plastic changes in brain micro-and macro- structure accompany functional abnormalities (53, 82–84). However, whatever the origin of these cerebral morpho-functional abnormalities, migraine manifestation requires ignition of the central and peripheral trigeminal system (2). Overt or silent cortical spreading depression (85), malfunctioning descending pain control systems in the frontal cortex (86) and brainstem (53, 87), and abnormal thalamic control (83, 88, 89)- alone or in combination- seem to be major permissive interictal factors for the preictal cascade of events that leads to sequential sensitization of first- and/or second-order trigeminovascular nociceptors resulting in transient (episodic migraine) or persistent (in CM) central sensitization (2, 90).

Here, we have summarized clinical, neurophysiological, and neuroimaging studies that have assessed the perception of pain in the various phases of the migraine cycle. The studies performed during the spontaneous occurrence of a migraine attack have detected lower PTs and increased PPI during the ictal phase compared to both the interictal phase and HC in response to various type of sensory stimuli applied over the cephalic region on the usual headache side. The involvement of ipsilateral and/or contralateral extracephalic territories depends mainly on the time passed from the beginning of an attack. These time and spatial changes in pain processing and sensitivity can be interpreted as reflecting subtle manifestations of the sequential activation of the peripheral and central sensitization processes, sometimes clinically manifesting as CA.

Contrary to what was observed during the ictal period, the results obtained during the interictal period are conflicting, as either reduced PT or no differences were observed. Moreover, PTs were unrelated to the presence of overt interictal/ictal CA, as well as the presence of CA was in any way related to PPI in response to different levels of stimulus intensity and induced only marginal changes in BOLD-fMRI signals. Despite these negative results, some studies show that migraine patients experience fluctuations in pain processing depending on the distance from the attack, as PT decreases preictally approaching the next attack, while PT greatly decreased and PPI increased ictally. Interestingly, a similar correlation with the number of days that had elapsed since the last attack was previously found in migraine with other sensory modalities, such as electrophysiology (91–94), psychophysical tests (95), and neuroimaging (53, 83, 96). Overall, these data indicate that a common neurobiological dysfunction might be responsible for abnormal information processing of both noxious and innocuous sensory stimuli. This interpretation is supported by the previous observation that the abnormal interictal processing of nociceptive blink reflex and innocuous visual evoked potentials are strictly interrelated when tested in the same patient between attacks (97).

As for the interictal state, PT data compared from patients evolving from episodic to CM to both episodic migraine patients and HCs are conflicting and inconclusive. Instead, higher PPI ratings were documented solely in CM patients overusing acute medications before drug withdrawal compared to HCs. The latter results may be explained as abnormal modulation of pain processing at the spinal level due to a decrease in antinociceptive activity of the supraspinal structures in MOH patients. This hypothesis is supported by recent neuroimaging studies showing altered structural integrity and functional connectivity of descending pain modulatory areas, such as periaqueductal gray (82, 98–100), and thalamic nuclei (101) in MOH patients.

Quantitative sensory testing (chiefly using LEPs) and functional neuroimaging studies in response to noxious stimuli show evidence of plastic reorganization in brain areas anchored to the so-called salience network (102), in response to the recurrence of migraine attacks and chronification, unrelated to the subjective PPI as assessed over the cephalic and extra-cephalic areas.

Future studies in this subject area in coming years should focus on the longitudinal investigation of a large group of patients before and after pharmacological and non-pharmacological interventions (103). This could help elucidate whether lower PT and increased pain sensitivity are primary risk factors or secondary consequences of migraine recurrence. To reduce discrepancies between studies, more attention should be given to the influence of chronic medication use on brain physiology, and the accurate collection of clinical data before, during and after the day of testing, to prospectively monitor the patients' clinical fluctuations, and be aware of changes resulting from increased consumption of acute medications and concomitant increase in attacks frequency.

## Author contributions

AR and GC: review the literature, manuscript drafting, and revision; GT, AT, and FP: manuscript revision; MS and VP: clinical and neurophysiological studies revision to perform Tables 1, 2. All authors read and approved the final manuscript.

### Conflict of interest statement

The authors declare that the research was conducted in the absence of any commercial or financial relationships that could be construed as a potential conflict of interest.
